# Draft Genome Sequence of *Corynebacterium pseudotuberculosis* Strain PA06 Isolated from a Subauricular Abscess in an Ovine Host

**DOI:** 10.1128/genomeA.00083-17

**Published:** 2017-03-30

**Authors:** Joana Montezano Marques, Vitória Almeida Gonçalves de Moura, Alyne Cristina Sodré Lima, Carla Thais Moreira Paixão, Amália Raiana Fonseca Lobato, Jorianne Thyeska Castro Alves, Ana Luiza de Mattos Guaraldi, Adriana Ribeiro Carneiro Folador, Rommel T. J. Ramos, Artur Silva

**Affiliations:** a Laboratory of Genomic and Bioinformatics, Federal University of Pará, Center of Genomics and System Biology, Belém, Pará, Brazil; b College of Medical Science, State University of Rio de Janeiro, Rio de Janeiro, Brazil

## Abstract

We report here the draft genome sequence of *Corynebacterium pseudotuberculosis* PA06, isolated from a subauricular abscess in an ovine host. *C. pseudotuberculosis* is a worldwide pathogen of small and large ruminants. The genome comprises 2,320,074 bp, with a G+C content of 52.2%, 2,195 coding sequences, 48 tRNAs, and three rRNAs.

## GENOME ANNOUNCEMENT

*Corynebacterium pseudotuberculosis* is a Gram-positive, nonmotile, non-spore-forming, pleomorfic, intracellular, and aerobic-facultative bacterium (1). This bacterium is the etiological agent of caseous lymphadenitis (CLA) disease in small ruminants. Besides its veterinary interest, this pathogen is a zoonotic agent and may be associated with many diseases distributed worldwide (2). The pathogenic effects of this agent have been observed in different hosts, including ulcerative lymphangitis in horses and ulcerative dermatitis in cattle. Recently, *C. pseudotuberculosis* has also been reported in pigs (3), and, by molecular genetic evidence, it was demonstrated that a veterinary student had pneumonia caused by this pathogen (4).

The primary pathology of this bacterium, caseous lymphadenitis, is a contagious disease that is characterized by the presence of abscesses in the lymph nodes and internal host organs (2). Considering the difficulties in identifying the disease’s early stages and the presence of antigens produced by infectious agents (5), caseous lymphadenitis presents itself as a difficult disease to eradicate. Thus, it is responsible for large economic losses by reducing wool quality, decreasing milk production, lowering weight, and causing death and damage to carcasses (2).

*C. pseudotuberculosis* PA06 was obtained from a caseous secretion from a punctured lymph node in the subauricular region of a sheep host (Santa Inês breed) located on a farm in Pará State, Brazil. The draft genome was sequenced using the Ion Torrent Personal Genome Machine (PGM) platform (Thermo Fisher) with a fragment library. The genome assembly was performed by SPAdes version 3.9.0 (6) with *k*-mers of 31, 33, 35, 61, 63, and 65, resulting in 20 contigs. Additionally, MIRA version 4.0 (7) was used with default parameters to reduce the assembly to seven contigs. MAUVE version 2.4 (8) was used to generate the scaffold that was automatically annotated with RAST version 2.0 (9). The genome has 2,195 coding sequences, three rRNAs, 48 tRNAs, and a G+C content of 52.2%.

### Accession number(s).

This whole-genome shotgun project has been deposited in GenBank under the accession number CP019289.
